# Activation of the PhoPR-Mediated Response to Phosphate Limitation Is Regulated by Wall Teichoic Acid Metabolism in *Bacillus subtilis*

**DOI:** 10.3389/fmicb.2018.02678

**Published:** 2018-11-06

**Authors:** Kevin M. Devine

**Affiliations:** Smurfit Institute of Genetics, Trinity College Dublin, Dublin, Ireland

**Keywords:** phosphate limitation, PhoBR *Escherichia coli*, PhoPR *Bacillus subtilis*, control of PhoR activity, phosphate transport mechanism, wall teichoic acid metabolism mechanism

## Abstract

Phosphorous is essential for cell viability. To ensure an adequate supply under phosphate limiting conditions, bacteria induce a cohort of enzymes to scavenge for phosphate, and a high affinity transporter for its uptake into the cell. This response is controlled by a two-component signal transduction system named PhoBR in *Escherichia coli* and PhoPR in *Bacillus subtilis*. PhoR is a sensor kinase whose activity is responsive to phosphate availability. Under phosphate limiting conditions, PhoR exists in kinase mode that phosphorylates its cognate response regulator (PhoB, PhoP). When activated, PhoB∼P/PhoP∼P execute changes in gene expression that adapt cells to the phosphate limited state. Under phosphate replete conditions, PhoR exists in phosphatase mode that maintains PhoB/PhoP in an inactive, non-phosphorylated state. The mechanism by which phosphate availability is sensed and how it controls the balance between PhoR kinase and phosphatase activities has been studied in *E. coli* and *B. subtilis*. Two different mechanisms have emerged. In the most common mechanism, PhoR activity is responsive to phosphate transport through a PstSCAB/PhoU signaling complex that relays the conformational status of the transporter to PhoR. In the second mechanism currently confined to *B. subtilis*, PhoR activity is responsive to wall teichoic acid metabolism whereby biosynthetic intermediates can promote or inhibit PhoR autokinase activity. Variations of both mechanisms are found that allow each bacterial species to adapt to phosphate availability in their particular environmental niche.

## Introduction

Phosphorous containing biomolecules participate in a wide range of cellular activities, including information processing, energy metabolism, signaling, regulation of protein activity, and maintenance of acid-base homeostasis. Furthermore, the cell envelopes of some bacteria (e.g., *Bacilli* and *Staphylococci*) contain lipoteichoic acid (LTA) and wall teichoic acid (WTA), anionic polymers with a high phosphorous content. LTA is a polymer of glycerol phosphate that extends into the cell wall from a lipid anchored in the cell membrane. WTA is a polymer of glycerol- or ribitol-phosphate that is covalently attached to peptidoglycan (Weidenmaier and Peschel, 2008; Percy and Gründling, 2014). These anionic polymers play important roles in cellular morphology and cell division (Swoboda et al., 2010; Percy and Gründling, 2014).

An adequate supply of phosphorous is therefore a prerequisite for cell viability. This is a challenge for bacteria especially those in habitats such as soil where the level of free phosphate can be low due to pH dependent formation of precipitates (Batjes, 1997). Bacteria adopt two general strategies to maintain an adequate phosphorous supply: (i) intracellular storage and (ii) scavenging for phosphate with enzymes induced under phosphorous limiting conditions. Many bacteria store phosphorous as polyphosphate, a polymer of hundreds of phosphate residues linked by high energy phosphoanhydride bonds that is synthesized by polyphosphate kinase (PPK1) using nucleotide triphosphates (Brown and Kornberg, 2008; Rao et al., 2009; Jiménez et al., 2017). Phosphate is released from the polyphosphate store by degradation with exo- and endo-polyphosphate phosphatases (Brown and Kornberg, 2008; Achbergerová and Nahálka, 2011). However, *Bacillus subtilis* subspecies *subtilis* (hereafter called *B. subtilis*) cannot synthesize polyphosphate but instead uses WTA as the store from which phosphate is released by the combined activities of the GlpQ and PhoD phosphodiesterases, whose expression is increased upon phosphate limitation in a PhoPR dependent manner (Eder et al., 1996; Antelmann et al., 2000; Myers et al., 2016).

Adaptation to phosphate limitation is often mediated by a two-component signal transduction system (TCS) named PhoBR in *Escherichia coli* and PhoPR in *B. subtilis* (hereafter called the PHO response). PhoR is a sensor kinase whose activity is responsive to phosphate availability. When activated, PhoR phosphorylates its cognate response regulator (PhoB or PhoP) that in turn directs a program of gene expression to adapt bacteria to the phosphate limited state. The PHO responses of these bacteria are characterized by (i) increased expression and secretion of enzymes that scavenge for phosphate; (ii) increased expression of a high affinity ABC-type phosphate transporter (PstSCAB), and (iii) amplification of the response by positive autoregulation of the *phoBR* and *phoPR* operons.

How PhoR senses phosphate and how its activity is controlled in a manner that is responsive to phosphate availability has been investigated mainly in *E. coli* and *B. subtilis*. Two different mechanisms have emerged that control the balance between the autokinase and phosphatase activities of PhoR. The first mechanism, exemplified by PhoBR activation in *E. coli*, is responsive to phosphate transport and is mediated by the PstSCAB transporter and PhoU adaptor protein (Hsieh and Wanner, 2010). The second mechanism, exemplified by PhoPR in *B. subtilis*, is responsive to WTA metabolism and is mediated by intermediates in WTA biosynthesis (Botella et al., 2014; Prunty et al., 2018). Variation of both mechanisms is observed that enable bacteria to ensure an adequate phosphorous supply in their particular ecological niches.

## Activation of PhoBR Is Responsive to Phosphate Transport by PstSCAB in *E.* *coli*

**FIGURE 1:**
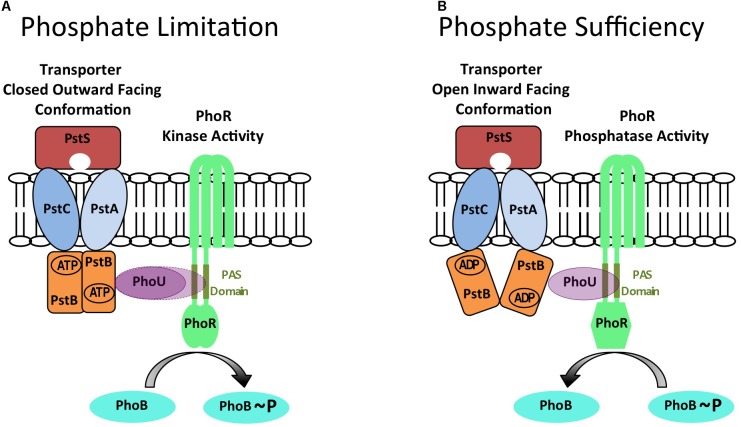
A model for controlling the balance between PhoR autokinase and phosphatase activities in *E. coli* generated by conformational changes in the PstSCAB phosphate transporter. The PhoR protein can exist in either autokinase mode during conditions of phosphate limitation **(A)** or in phosphatase mode during conditions of phosphate sufficiency **(B)**. It is proposed that these alternate states are determined by different conformations of the PstSCAB transporter that are relayed to PhoR by PhoU to determine its activity. When PstSCAB is in a closed outward facing conformation **(A)** the PhoU adaptor protein is either unable to interact with PhoR (PhoU solid lines), or interacts with the PAS domain of PhoR to promote autokinase activity (PhoU broken lines) during conditions of phosphate limitation. The cognate PhoB response regulator is phosphorylated in this condition. However, when PstSCAB is in an open inward facing conformation **(B)** the PhoU adaptor protein interacts with the PAS domain of PhoR to promote phosphatase activity during conditions of phosphate sufficiency. The cognate PhoB response regulator is dephosphorylated in this condition. This diagram is an adaptation of the models and figures presented in Hsieh and Wanner (2010) and Vuppada et al. (2018).

Seven proteins are necessary and sufficient for induction of the PHO response in *E. coli*: the PhoBR TCS, the PstSCAB ABC-type phosphate transporter and the PhoU protein that is encoded in the *pstSCABphoU* operon (Hsieh and Wanner, 2010). PhoBR is a classical TCS. PhoR is membrane associated and composed of two transmembrane domains and a cytoplasmically located PAS (Per Arndt Sim) domain that interacts with PhoU. The PhoB response regulator is activated by PhoR-mediated phosphorylation and binds to PHO boxes located in the promoter region of PhoBR regulon genes (Blanco et al., 2002). The PstSCAB ABC-type transporter is composed of a periplasmically located PstS protein that binds phosphate, PstCA proteins located within the membrane that form the transport channel and a PstB dimer that binds ATP and provides energy for the transport process. The PhoU protein is a membrane-associated metal binding protein composed of two three-alpha helical bundles that forms multimers (Liu et al., 2005; Oganesyan et al., 2005). Genetic analysis has shown that the PHO response is constitutively activated when either the PstSCAB transporter or PhoU is deleted (Figure 1A; Hsieh and Wanner, 2010). This implies that PhoR is in default autokinase mode under phosphate limiting conditions and that the PstSCAB high affinity transporter and PhoU are both required for its conversion to phosphatase mode under phosphate replete conditions (Hsieh and Wanner, 2010). The PhoU protein interacts with the PAS domain of PhoR and the PstB protein of the PstSCAB transporter to form a membrane bound signaling complex that converts PhoR to phosphatase activity mode (Gardner et al., 2014, 2015). The PhoU protein also modulates the activity of the PstSCAB transporter (Rice et al., 2009). Results show that the balance between the autokinase and phosphatase modes of PhoR activity is controlled neither by intracellular phosphate concentration nor by transport of phosphate *per se* (Cox et al., 1988, 1989; Rao et al., 1993). Instead, there is evidence from a genetic approach stabilizing PstB in two different conformations, that conformational changes in the PstSCAB transporter are important in determining the balance between the autokinase and phosphatase modes of PhoR activity (Vuppada et al., 2018). When the transporter is present in an outward facing ‘closed’ structure, the PstSCAB/PhoU signaling complex either does not interact with PhoR (Figure 1A, PhoU solid lines) or interacts with PhoR (Figure 1A, PhoU broken lines) to promote autokinase activity, the state that exists under phosphate limiting conditions. However, when the transporter is present in an inward facing ‘open’ structure, the PstSCAB/PhoU signaling complex interacts with PhoR to promote phosphatase activity, the state that exists under conditions of phosphate sufficiency (Figure 1B). Thus phosphate availability in *E. coli* is sensed indirectly through conformational changes in the PstSCAB transporter (Gardner et al., 2015; Vuppada et al., 2018). In fact, transporters have now been shown to have a signaling role in controlling the activity of several TCS (for reviews see Tetsch and Jung, 2009; Piepenbreier et al., 2017).

## Variations of the PhoBR/PstSCAB/PhoU Regulatory Theme Present in Other Bacteria

The PhoBR/PstSCAB/PhoU theme of regulation is widely used to control phosphate metabolism and related cellular processes (e.g., virulence) in bacteria (Lamarche et al., 2008; Chekabab et al., 2014; Santos-Beneit, 2015). However, the theme can be varied by alteration of the number, activities or interactions of the constituent components as illustrated by these selected examples. In *Caulobacter crescentus*, PhoU is an essential protein that does not control PhoR activity but regulates intracellular phosphate metabolism (Lubin et al., 2015). There are two PhoU proteins encoded in *Staphylococcus epidermidis*: the *phoU1* gene is located within the *pstSCABphoU1* operon while *phoU2* is located upstream of a gene with homology to the PitA phosphate transporter (Wang et al., 2017). Genetic analysis indicates that only PhoU2 controls PhoPR activity (Wang et al., 2017). *Streptococcus pneumoniae* encodes a single two-component system PnpRS located upstream of a phosphate transporter Pst1 (*pstS1C1A1B1phoU1*) but encodes a second phosphate transporter Pst2 (*pstS2C2A2B2phoU2*) at a distinct chromosomal locus (Zheng et al., 2016). Expression of Pst2 is constitutive and PhoU2 inhibits Pst2 transporter activity. Expression of Pst1 is induced by phosphate limitation in a PnpRS dependent manner while PhoU1 inhibits Pst1 transporter activity (Zheng et al., 2016). However, PhoU2, but not PhoU1, controls PnpRS activity in a manner similar to that in *E. coli* (Zheng et al., 2016). These selected examples indicate the versatility of the PhoBR/PstSCAB/PhoU regulatory theme and show how it can be varied in bacteria to respond to phosphate availability in their particular ecological niche.

## Activation of PhoPR in *B. subtilis* Is Responsive to Wall Teichoic Acid Metabolism

**FIGURE 2:**
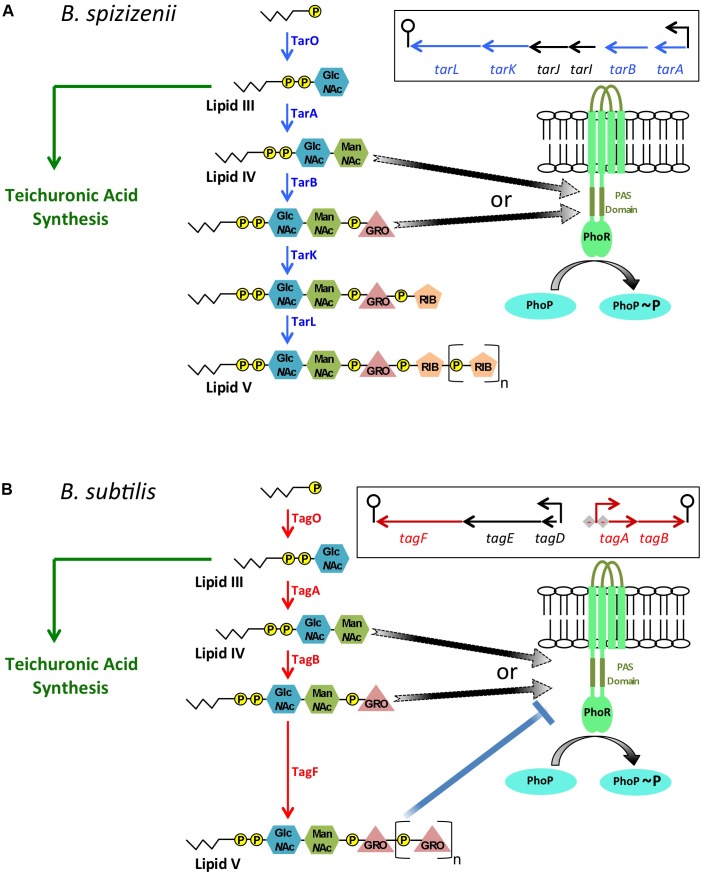
A model for controlling the balance between PhoR autokinase and phosphatase activities in *B. subtilis* and *B. spizizenii* generated by intermediates in wall teichoic acid metabolism. In *B. subtilis* and *B. spizizenii* it is proposed that autokinase and phosphatase modes of PhoR activity are determined by WTA biosynthetic intermediates levels. **(A)** The metabolic pathway for WTA biosynthesis in *B. spizizenii* is shown with the final Lipid V product being composed of poly(ribitol phosphate) attached to a lipid carrier (jagged lines) by a linker composed of pyrophosphate, modified sugars (Glc*N*Ac, Man*N*Ac) and a single glycerol phosphate moiety. The genomic organization of the biosynthetic genes is shown in the box. It is proposed that the TarA or TarB enzyme product activates PhoR autokinase activity (arrows with broken lines to indicate a putative interaction). The level of activating intermediate will be determined by the balance between WTA and teichuronic acid synthesis in phosphate limited cells because of the reciprocal relationship between these two metabolic pathways. **(B)** The metabolic pathway for WTA biosynthesis in *B. subtilis* is shown with the final Lipid V product being composed of poly(glycerol phosphate). The genomic organization of the biosynthetic genes (in box) shows that the *tagAB* operon is repressed by activated PhoP∼P (-). It is proposed that the TagA or TagB enzyme product activates PhoR autokinase activity (arrows with broken lines). However, in *B. subtilis* Lipid V composed of poly(glycerol phosphate) can inhibit PhoR autokinase activity (shown *in vitro*, blue line with block). Thus the balance between PhoR autokinase and phosphatase activities will be determined by the level of the activating intermediates (TagA or TagB, black arrows), by the extent to which teichuronic acid synthesis is activated and WTA synthesis is repressed by PhoP∼P and by the level of the inhibitory Lipid V composed of poly(glycerol phosphate), blue line with block).

Several features of the PHO response in *B. subtilis* indicate that it differs from that of *E. coli*. The *B. subtilis* genome does not encode a PhoU homologue and the PHO response is induced normally in a strain with the high-affinity phosphate transporter (*pstSCAB_1_B_2_*) deleted (Qi et al., 1997). Furthermore unlike *E. coli*, the composition and metabolism of cell wall anionic polymers is changed in a PhoPR-dependent manner during the PHO response in *B. subtilis* (Liu and Hulett, 1998; Liu et al., 1998; Allenby et al., 2005; Botella et al., 2011, 2014; Salzberg et al., 2015). When activated, PhoP∼P represses transcription of the *tagAB* operon thereby reducing WTA synthesis, and activates transcription of the *tuaA-H* operon thereby increasing synthesis of teichuronic acid, a replacement non-phosphate containing anionic polymer (Figure 2; Liu and Hulett, 1998; Liu et al., 1998; Allenby et al., 2005; Botella et al., 2011). In addition, PhoPR regulon genes encode a plethora of enzymes for scavenging phosphate including the GlpQ and PhoD posphodiesterases that function in WTA degradation (Eder et al., 1996; Antelmann et al., 2000; Allenby et al., 2005; Botella et al., 2011; Myers et al., 2016). Furthermore, *B. subtilis* is unable to synthesize polyphosphate.

PhoR in *B. subtilis* also differs from that in *E. coli* in having an extracytoplasmic loop capable of forming a PAS domain that is not required for induction of the PHO response under laboratory conditions (Figure 2; Shi and Hulett, 1999; Chang et al., 2010; Botella et al., 2014). Amplification of the PHO response by positive autoregulation of *phoPR* transcription is augmented by PhoP∼P binding to PhoP-boxes located between the *phoR* stop codon and the operon terminator, by an as yet unknown mechanism (Salzberg et al., 2015). It is perhaps not surprising therefore that activation of the PHO response in *B. subtilis* differs from that in *E. coli* and that it involves WTA metabolism.

The link between anionic polymer metabolism and PhoR activity was established in *B. subtilis* by the finding that amplification of the PHO response is delayed and attenuated in cells unable to synthesize teichuronic acid (Botella et al., 2014). Since transcription of the *tuaA-H* operon requires activated PhoP∼P, this result indicates that teichuronic acid biosynthesis amplifies the PHO response by a positive feedback mechanism to increase PhoR autokinase activity (Botella et al., 2014). This observation is especially significant since WTA synthesis is reduced by PhoP∼P mediated repression of *tagAB* expression. Furthermore, there is a reciprocal relationship between WTA and teichuronic acid synthesis in *B. subtilis* (the TagO enzyme product is a precursor in both metabolic pathways) implying that an increase in teichuronic acid synthesis will cause a decrease in WTA synthesis (Figure 2; Botella et al., 2014). Therefore, we sought to establish if increased PhoR activity during amplification of the PHO response is caused by an increase in the level of a teichuronic acid biosynthetic intermediate that promotes autokinase activity or by a decrease in the level of a WTA biosynthetic intermediate that inhibits autokinase activity. Genetic and biochemical evidence shows that (i) attenuation of the PHO response in strains unable to synthesize teichuronic acid can be suppressed by a concomitant reduction in WTA synthesis and (ii) PhoR autokinase activity is inhibited *in vitro* by a derivative of the WTA biosynthetic intermediate produced by the TagF enzyme (Botella et al., 2014). These data show that PhoR autokinase activity is responsive to WTA metabolism in *B. subtilis*, being inhibited by the product of the TagF enzyme (Botella et al., 2014).

Since the PHO response is amplified in *B. subtilis* by two processes that require activated PhoP∼P (i.e., reduction of WTA synthesis and activation of teichuronic synthesis), it is evident that it must be initiated by a different PhoP∼P-independent mechanism. Thus, initiation of the PHO response was addressed in *Bacillus subtilis* subspecies *spizizenii* (hereafter called *B. spizizenii*) that synthesizes WTA composed of poly(ribitol phosphate) (*tarABIJKL* genes) (Prunty et al., 2018). Investigation of the PHO response in *B. spizizenii* has the advantage that it is not amplified as in *B. subtilis* [i.e., WTA intermediates composed of poly(ribitol phosphate) do not inhibit PhoR autokinase activity], and expression of WTA biosynthetic enzymes is not repressed by PhoP∼P (Prunty et al., 2018). Three pieces of evidence indicate that the PHO response is activated by an intermediate in WTA synthesis in *B. spizizenii*. (1) In a strain with inducible expression of the *tarABIJKL* operon encoding the WTA biosynthetic enzymes, the magnitude of the PHO response is directly proportional to the level of added inducer (Prunty et al., 2018). (2) The PHO response is increased in a strain of *B. spizizenii* unable to synthesize teichuronic acid, indicating that the onset of teichuronic acid synthesis reduces the level of a WTA intermediate that activates the PHO response (Prunty et al., 2018). (3) The increased PHO response in a strain unable to synthesize teichuronic acid is reduced by lowering expression of the genes that encode the WTA biosynthetic enzymes (Prunty et al., 2018). Similar results were obtained in *B. spizizenii* strains expressing either the homologous PhoR kinase or the PhoR kinase from *B. subtilis* showing that activation occurs by the same mechanism in both subspecies (Prunty et al., 2018). These results suggest that an intermediate in WTA synthesis activates PhoR autokinase activity in both *B. subtilis* and *B. spizizenii* (Prunty et al., 2018). Since only the TagA/TarA and TagB/TarB enzymatic steps of the WTA biosynthetic pathways are common in *B. subtilis* and *B. spizizenii*, we conclude that one or other of their enzyme products promotes PhoR autokinase activity (Prunty et al., 2018).

A model for activation of the PHO response in *B. spizizenii* and *B. subtilis* (Figures 2A,B, respectively) proposes that upon phosphate limitation (i) there is a surge in the cellular level of WTA intermediates caused by a reduction in growth rate and lowered cell wall synthesis and (ii) the increased level of TagA/TarA or TagB/TarB enzyme product promotes PhoR autokinase activity (Prunty et al., 2018). In addition, PhoR autokinase activity is controlled in *B. subtilis* only by the level of the TagF enzyme product composed of poly (glycerol phosphate) (Figure 2B; Botella et al., 2014).

In summary, the balance of PhoR autokinase and phosphatase activities is responsive to WTA metabolism in *B. subtilis* and *B. spizizenii* (Prunty et al., 2018).

## Variations of the PhoPR/Wall Teichoic Acid Metabolism Regulatory Theme

The kinetics of the PHO responses of *B. subtilis* and *B. spizizenii* are different (Botella et al., 2014; Prunty et al., 2018). In *B. subtilis*, the PHO response is activated, then amplified and maintained while phosphate limitation persists (Botella et al., 2014). In *B. spizizenii* the PHO response is activated but then gradually turned off even with persistence of phosphate limiting conditions (Prunty et al., 2018). These separate responses are not due to differences in the PhoR kinases, which are highly homologous and experimentally shown to be functionally equivalent (Prunty et al., 2018). Instead, the different PHO responses derive from the fact that the TagF enzyme product composed of poly(glycerol phosphate) inhibits PhoR autokinase activity in *B. subtilis* but the corresponding TarL enzyme product composed of poly(ribitol phosphate) does not inhibit PhoR autokinase activity in *B. spizizenii* (Figure 2; Botella et al., 2014; Prunty et al., 2018). Therefore, host features that impact on expression of WTA biosynthetic enzymes and the level of WTA intermediates will influence the PHO responses of these two subspecies. These host features include genomic organization of WTA biosynthetic genes, regulation of their expression and their cellular mRNA level (Prunty et al., 2018). All WTA biosynthetic enzymes are encoded in a single operon (*tarABIJKL*) in *B. spizizenii* (Figure 2A) but are separated into two operons (*tagAB* and *tagDEF*) in *B. subtilis* (Figure 2B). Expression of the *tarABIJKL* operon is not regulated by PhoPR in *B. spizizenii* (Figure 2A) whereas expression of the *tagAB* operon is negatively regulated by activated PhoP∼P in *B. subtilis* (Figure 2B). In *B. spizizenii*, mRNA levels of the *tarABIJKL* genes are uniformly reduced to ∼15% of that found in exponentially growing phosphate replete cells (Prunty et al., 2018). However, in *B. subtilis*, mRNA levels of the *tagAB* genes are reduced to <1%, while that of *tagF* is only reduced to 27%, of that found in exponentially growing, phosphate replete cells. Thus, these different host features direct distinct PHO responses in *B. subtilis* and *B. spizizenii* by altering WTA metabolism, allowing adaptation to phosphate availability to be optimized in particular ecologicalniches.

## Concluding Remarks

The bacterial PHO response is widely conserved among bacteria. However, two different mechanisms have emerged by which PhoR activity is controlled in a manner that is responsive to phosphate availability. A mechanism responsive to the conformation of the PstSCAB phosphate transporter in conjunction with PhoU, exemplified by PhoBR in *E. coli*, that is widespread among Gram-positive and Gram-negative bacteria. A distinct mechanism that is responsive to WTA synthesis is exemplified by PhoPR in *B. subtilis*. It is unclear why such a distinct mechanism has evolved in *B. subtilis*, but it may relate to the high phosphate composition of cell wall anionic polymers, the ability to alter anionic polymer composition during phosphate limitation, the inability to synthesize polyphosphate and the use of WTA as a phosphate store. It is likely that PhoPR is activated in a PhoU-type mechanism in Staphylococcal species suggesting that phosphorous containing WTA is not a determining feature in evolution of the novel mechanism in *B. subtilis*. However, differences in phosphorous availability in their natural habits may have played a significant role, with its availability in soil being especially problematical for Bacillus species. Therefore it will be interesting to establish how stains of *B. subtilis* and *Staphylococcus aureus* expressing the PhoPR activation mechanism of the other bacterium adapt to phosphate limiting conditions and survive in their natural habitat.

## Autor Contributions

KD designed the review and drafted the manuscript.

## Conflict of Interest Statement

The authors declare that the research was conducted in the absence of any commercial or financial relationships that could be construed as a potential conflict of interest.
